# Nutritional knowledge, insulin resistance, and the risk of orthorexia nervosa: a comparative cross-sectional study among polish women

**DOI:** 10.3389/fpubh.2025.1562866

**Published:** 2025-03-19

**Authors:** Wiktoria Staśkiewicz-Bartecka, Karolina Masłoń, Aleksandra Kołodziejczyk, Agnieszka Białek-Dratwa, Agata Kiciak, Sylwia Jaruga-Sękowska, Daria Dobkowska-Szefer, Mateusz Grajek, Oskar Kowalski, Marek Kardas

**Affiliations:** ^1^Department of Food Technology and Quality Evaluation, Department of Dietetics, Faculty of Public Health in Bytom, Medical University of Silesia in Katowice, Zabrze, Poland; ^2^Department of Human Nutrition, Department of Dietetics, School of Public Health in Bytom, Medical University of Silesia in Katowice, Zabrze, Poland; ^3^Department of Health Promotion, Faculty of Public Health in Bytom, Medical University of Silesia in Katowice, Bytom, Poland; ^4^Department of Public Health, Department of Public Health Policy, Faculty of Public Health in Bytom, Medical University of Silesia in Katowice, Bytom, Poland

**Keywords:** orthorexia nervosa, insulin resistance, nutritional knowledge, women, mental health

## Abstract

**Background:**

The global rise in insulin resistance has led to an increased emphasis on dietary modifications as a primary strategy for its management. While such interventions are essential for improving metabolic health, they can also contribute to heightened nutritional knowledge. However, this increased focus on diet may inadvertently lead to the development of disordered eating patterns, including orthorexia nervosa. This study aimed to determine the level of nutritional knowledge regarding proper eating habits among women with insulin resistance and to assess the relationship between this knowledge and the risk of developing orthorexia nervosa.

**Methods:**

The study was using the Computer-Assisted Web Interview method, involving 133 female participants from a primary care clinic in Katowice, Poland. Of these, 101 women had a medically confirmed diagnosis of insulin resistance, and 32 were healthy controls. Data were collected using an online survey, which included a demographic section, a 15-item questionnaire to assess nutritional knowledge, and the ORTO-15 tool to evaluate orthorexia nervosa risk.

**Results:**

Women with insulin resistance had significantly higher Body Mass Index values and demonstrated greater nutritional knowledge than their healthy counterparts. However, 56.44% of women with insulin resistance were at risk of orthorexia nervosa, compared to 15.63% of the control group. Increased dietary knowledge in women with insulin resistance was also associated with a higher risk of developing orthorexic behavior.

**Conclusion:**

The findings indicate that while women with insulin resistance benefit from improved nutritional knowledge in managing their condition, this knowledge may simultaneously increase their risk of developing orthorexia nervosa. Balancing the promotion of healthy eating habits with strategies that prevent the emergence of distorted eating behaviors is crucial. Future interventions should emphasize flexibility, psychological support, and individualized guidance to ensure both metabolic and mental well-being.

## 1 Introduction

Today’s lifestyle, characterized by haste, lack of time, and neglect of proper nutrition, can contribute to the increasing prevalence of insulin resistance (IR). IR, a metabolic disorder, is closely associated with reduced physical activity and excess body fat (1, 2). It is a condition that precedes the onset of type II diabetes and can also lead to the development of other chronic diseases (3, 4). According to the International Diabetes Federation (IDF), in 2021, approximately 537 million adults aged 20–79 worldwide were affected by diabetes (5). In Poland, data from the National Health Fund (NFZ) showed that in 2018, 2.6 million people (9.1% of the adult population) suffered from diabetes (6). This condition most commonly affects individuals with overweight and obesity who do not follow a well-balanced diet and have low physical activity levels; however, genetic factors may also play a role (2). The rapid global increase in IR poses significant health and economic burdens (7).

Dietary intervention is the primary strategy for managing IR, emphasizing the importance of nutritional awareness and adherence to dietary recommendations (8). However, excessive focus on healthy eating can lead to other issues, such as orthorexia nervosa (ON). ON is not classified as an eating disorder (ED) in the major systems, the ICD-10 (International Classification of Diseases) and the DSM-5 (Diagnostic and Statistical Manual of Mental Disorders) (9, 10). The term was first introduced in 1997 by physician S. Bratman to describe a pathological obsession with healthy eating (11). The key feature of ON is the pursuit of optimal health through rigid control over the quality of consumed food (12). Individuals affected by this disorder obsessively focus on the quality of their diet, and deviations from self-imposed dietary rules lead to anxiety and guilt, resulting in even stricter eating behaviors. Consequences of ON include malnutrition, excessive weight loss, and difficulties in social functioning, such as avoidance of social gatherings due to anxiety surrounding food discussions (13, 14).

Research conducted over the past decade indicates a growing number of people suffering from ON. This condition most frequently affects young individuals, particularly those with higher education who are highly concerned with their health. The risk of ON in the general population is estimated at 7%, but it is higher among individuals who follow strict dietary plans or have dietary restrictions (15, 16). It is also hypothesized that ON may be linked to significant anxiety about illness or death, with hypochondriacal tendencies playing a substantial role in its development (17, 18).

Given the growing prevalence of both IR and ON, understanding the relationship between these two conditions is crucial for improving health outcomes. IR often requires strict dietary modifications and weight control, which may predispose individuals to develop obsessive behaviors regarding food. Exploring these connections can provide valuable insights for healthcare professionals to implement balanced and sustainable dietary interventions promoting physical and mental well-being. Additionally, further research into the shared risk factors and behavioral patterns underlying IR and ON could help identify vulnerable groups and develop targeted educational programs to mitigate these health challenges.

Emerging research suggests that ON often develops in individuals who already adhere to strict dietary regimens, particularly those managing health conditions such as IR. A recent qualitative study by Tragantzopoulou and Giannouli (18) found that individuals experiencing health-related anxiety may adopt restrictive eating patterns as a coping mechanism to regain control over their health. This highlights the psychological dimension of ON and its potential to manifest in populations where dietary regulation is medically advised. The overlap between dietary restrictions for managing IR and the restrictive eating patterns observed in ON underscores the need to examine the psychological implications of dietary interventions for individuals with IR.

The rising global prevalence of IR presents a significant public health challenge, with direct implications for the prevention of type 2 diabetes and other metabolic disorders. While existing literature extensively documents the physiological and dietary interventions necessary to manage IR, limited attention has been given to the psychological and behavioral consequences of heightened nutritional knowledge in affected individuals. Previous studies have primarily focused on the benefits of dietary education and adherence to nutritional guidelines for IR management, but few have explored the potential for this knowledge to contribute to maladaptive eating patterns, such as ON. This study aims to bridge this gap by investigating the relationship between nutritional knowledge and the risk of ON in women with IR, a novel approach that reflects the growing need to balance physical health with psychological well-being. By addressing the interplay between metabolic disorders and disordered eating behaviors, this research offers new insights into the dual burden of IR and ON, highlighting the importance of integrated, holistic healthcare strategies.

This study aimed to determine the level of nutritional knowledge regarding proper eating habits among women with insulin resistance, and to assess the relationship between this knowledge and the risk of developing ON. The hypothesis posits that women with IR who demonstrate heightened nutritional awareness are at a greater risk of developing ON. Women with metabolic disorders such as IR may adopt strict dietary regimens as a way to exert control over their health, further increasing the risk of developing an eating disorder. This research contributes to the growing literature on the psychological impacts of managing metabolic conditions and underscores the importance of integrating mental health considerations into dietary interventions.

## 2 Materials and methods

### 2.1 Procedure for the study

The study was conducted in January–March 2024 using the CAWI (Computer-Assisted Web Interview) method, a widely accepted approach in psychological and behavioral research due to its efficiency, broad reach, and ability to maintain respondent anonymity (17). Data were collected via the Google Forms platform, chosen for its accessibility, user-friendly interface, and automated data aggregation, which streamlined subsequent data analysis.

A structured online questionnaire, comprising a total of 43 items, was disseminated via a dedicated URL link. This link was made available to female patients of a selected primary care clinic in Katowice, Poland. Before completing the questionnaire, participants were informed about the study’s purpose, the anonymity of their responses, and the voluntary nature of their participation. Such procedures aimed to minimize the risk of systematic error and reduce response bias.

A purposive sampling method was employed to ensure that the sample reflected the specific characteristics relevant to the study objectives—namely women with a medically confirmed diagnosis of IR and healthy women with no chronic conditions.

The sample selection for the study was based on a purposive recruitment scheme that allowed us to focus on women with confirmed IR. Recruitment for the study had a multi-stage approach. In the first stage, outpatients were informed about the study during follow-up visits, and the clinic used its internal communication platforms, including email newsletters and social media channels, to disseminate information about the study. Female participants willing to participate in the study were given a link to an online questionnaire prepared in Google Forms. For the control group, the recruitment process followed an analogous procedure to minimize environmental differences and achieve data comparability.

Before data collection, participants were informed about the study’s objectives, voluntary nature, and their rights to anonymity and withdrawal without consequences. This information was clearly outlined on the first page of the questionnaire, and informed consent was obtained from all participants before proceeding with the survey. The study was conducted in accordance with the principles of the World Medical Association’s Declaration of Helsinki. Ethical approval was obtained from the Bioethics Committee of the Medical University of Silesia in Katowice (PCN/0022/KB/299/19/20; approval date: 21.01.2020), pursuant to the Law of December 5, 1996 on the profession of physician and dentist (Journal of Laws 2016, item 727).

The sample size was estimated using a standard formula for the minimum required sample size, considering the number of female patients with IR regularly visiting a selected primary care clinic in Katowice. Of the women invited, 105 met the inclusion criteria. Finally, after excluding 4 incomplete or erroneously completed questionnaires, 101 complete responses were included in the analysis. The number of women with identified IR for a minimum of 1 year at the clinic was 140. The following formula was applied in the study:


$$N_{\text{min}}=\frac{N_{P}\left(\alpha^{2}.f\left(1-f\right)\right)}{N_{P}.e^{2}+\alpha^{2}.f\left(1-f\right)}$$

Where: 𝑁_𝑚𝑖𝑛_, minimum sample size; 𝑁_𝑃_, population size; 𝛼, confidence level; 𝑓, fraction size; 𝑒, assumed maximum error.

### 2.2 Participants

A total of 133 women participated in the study. Among them, 101 (75.94%) were medically diagnosed with insulin resistance, while 32 (24.06%) formed the control group—healthy women without chronic illnesses and not requiring long-term medication. All participants took part voluntarily. They were informed of the study’s objectives, assured of their anonymity, and reminded of their right to withdraw at any stage.

Inclusion criteria for the study were as follows: (1) providing informed consent to participate in the study, (2) being 18 years of age or older, (3) having IR diagnosed by a physician a minimum of 1 year ago (study group), (4) holding patient status at the selected primary care clinic. The diagnosis was based on standard biochemical tests, including fasting plasma glucose (FPG) and one additional test: the oral glucose tolerance test (OGTT) and the homeostasis model of insulin resistance (HOMA-IR). Cut-off points used for diagnosis included fasting glucose levels ≥100 mg/dL, OGTT results ≥140 mg/dL after 2 h and HOMA-IR values above 2.5, indicating impaired insulin sensitivity.

Exclusion criteria were: (1) absence from a visit during the study period, (2) incorrect or incomplete questionnaire completion, (3) diagnosed psychiatric disorders (such as depression, eating disorders, or anxiety), (4) diagnosed metabolic or hormonal conditions other than insulin resistance (e.g., polycystic ovary syndrome (PCOS), thyroid disorders), (5) use of medications known to affect appetite or food intake, including antipsychotics, corticosteroids, or weight-loss drugs. The control group consisted of women who were healthy and not taking long-term medication, and who also visited the same primary care clinic during the study period.

To minimize the influence of potential confounders, women from a single primary care clinic were included in the study; in addition, participants with diagnosed psychiatric disorders and metabolic and endocrine diseases other than insulin resistance were excluded. In addition, women taking medication that may affect appetite or food intake were excluded.

### 2.3 Research tools

The study employed a survey questionnaire consisting of a demographic section (age, weight, and height, place of residence, education, IR diagnosis, medication use, adherence to medical recommendations, comorbidities, supplement intake) and a set of questions assessing nutritional knowledge, along with the ORTO-15 questionnaire.

#### 2.3.1 Body mass index (BMI)

The nutritional status of the participants was assessed by body mass index, calculated according to the formula:


$$BMI=\frac{\mathrm{body}\;\mathrm{weight}\;\left(\mathrm{kg}\right)}{\mathrm{height}\;{\left(m\right)}^{2}}$$

The results were interpreted according to the World Health Organization (WHO) guidelines (19).

#### 2.3.2 Nutritional knowledge

Nutritional knowledge was assessed using an original set of 15 single-choice questions focused on key issues related to insulin resistance: its symptoms, dietary management strategies, glycemic index and load, and appropriate food selection. Each correct response was awarded one point. Based on the number of correct answers, three levels of nutritional knowledge were identified:

- Insufficient nutritional knowledge - ≤ 6 pkt. (≤ 40%).
- Sufficient nutritional knowledge - 7-11 pkt. (41–79%).
- Excellent nutritional knowledge - 12-15 pkt. (80–100%).

The validation of this original questionnaire involved a multi-step process to ensure its content validity, clarity, and internal consistency. First, an expert panel consisting of dietitians, nutritionists, and clinicians specializing in metabolic disorders reviewed the initial pool of items. This expert review led to refining certain questions to ensure their relevance to IR and the accuracy of their content.

A pilot study was then conducted on a group of 15 participants representative of the target population. Participants provided feedback on the clarity, comprehensibility and perceived difficulty of the questions. Based on their feedback, minor linguistic and structural adjustments were made to improve readability and interpretability.

A Cronbach’s alpha coefficient was calculated for the final version of the questionnaire to assess internal consistency. The resulting alpha coefficient of 0.711 indicated an acceptable level of reliability.

#### 2.3.3 Orto-15

The ORTO-15 Questionnaire in its Polish validation (20) was used in this study to assess the risk of ON. This tool is widely used in epidemiological and clinical studies to assess dietary patterns associated with the risk of ON.

The ORTO-15 Questionnaire consists of 15 questions addressing eating habits, attitudes toward food, and emotional reactions related to meal consumption. Each question employs a Likert scale with four response options: always, often, sometimes, and never. Participants’ responses are assigned scores, where lower scores indicate a higher risk of orthorexia (20, 21).

A score of ≤35 points suggests the presence of ON, while a score of >35 points indicates no risk.

The Polish validation of the ORTO-15 Questionnaire takes into account the linguistic and cultural specificity of the Polish population, ensuring the tool’s reliability and validity. The instrument was adapted following psychometric validation guidelines, providing a robust and reliable measure for assessing orthorexic tendencies within the study group (20). The internal consistency of the Polish version of the ORTO-15, measured using Cronbach’s omega coefficient, is 0.79, indicating an acceptable level of reliability for the scale.

### 2.4 Statistical analysis

Statistical analyses were conducted using Statistica v.13.3 (Stat Soft, Kraków, Poland) and the R package v. 4.0.0 (2020) under the GNU GPL (The R Foundation for Statistical Computing). Quantitative data were presented as mean ± standard deviation (X ± SD), while qualitative data were expressed as percentages. The Shapiro–Wilk test was applied to assess the normality of data distribution. For comparisons between two groups, the Student’s *t*-test was used for normally distributed data, whereas the Mann–Whitney *U* test was employed for non-normal distributions. Fisher’s exact test and the Chi-square (*χ*^2^) test were used to evaluate associations between categorical variables, with the strength of these relationships measured using Cramér’s V and Yule’s *φ* coefficients. Correlations between continuous variables were analyzed through Pearson’s correlation test. Additionally, linear regression analysis was performed to identify predictors of ORTO-15 scores, examining the relationship between dietary knowledge, BMI, and the presence of insulin resistance. Statistical significance was determined at a threshold of *p* < 0.05.

## 3 Results

### 3.1 Sample characteristics

The table below shows the characteristics of the study group of women (*N* = 133; 75.94%), including women with IR (WIR) (*N* = 101; 24.06%) and healthy women (CG) (*N* = 32). The mean age of all respondents was 39.90 ± 14.90. The youngest respondent was 19 years old and the oldest was 74 years old. In the study group, women were most likely to have secondary and tertiary education, with 42.86% (*N* = 57) and 42.10% (*N* = 56) respectively; no significant difference was found between groups (*p* = 0.185). Medication prescribed by the treating physician for IR was used by more than half of the respondents diagnosed with the disorder (53.47%; *N* = 54). The vast majority of women with IR 71.29% (*N* = 72) declared that they followed their doctor’s/dietitian’s recommendations in terms of diet, while 23.76% (*N* = 24) of respondents followed this sometimes and 4.95% (*N* = 5) did not at all. The vast majority of respondents, as many as 81.95% (*N* = 109), reported using dietary supplements, 78.12% (*N* = 25) of healthy women and 83.17% (*N* = 84) of women with insulin resistance. No significant differences were found between the groups (*p* = 0.518). The characteristics of the study group are shown in Table 1.

**Table 1:** Characteristics of the study group (*n* = 133).

	AP *N* = 133 X ± SD	WIR *N* = 101 X ± SD	CG *N* = 32 X ± SD	*p*-value	ε^2^
Age [years]	39.90 ± 14.90	39.65 ± 13.92	40.69 ± 17.88	0.734	0.00
Height [cm]	165.84 ± 6.88	165.58 ± 6.93	166.66 ± 6.73	0.444	0.00
Body Weight [kg]	73.88 ± 16.73	77.81 ± 16.68	61.47 ± 9.20	<0.001*	0.21
BMI [kg/m^2^]	26.81 ± 5.42	28.30 ± 5.16	22.12 ± 3.01	<0.001*	0.29

AP, All Participants; WIR, women with insulin resistance; CG, control group; *, *p* < 0.05; X, average; SD, standard deviation; ε^2^, Effect sizes.

**Figure 1:**
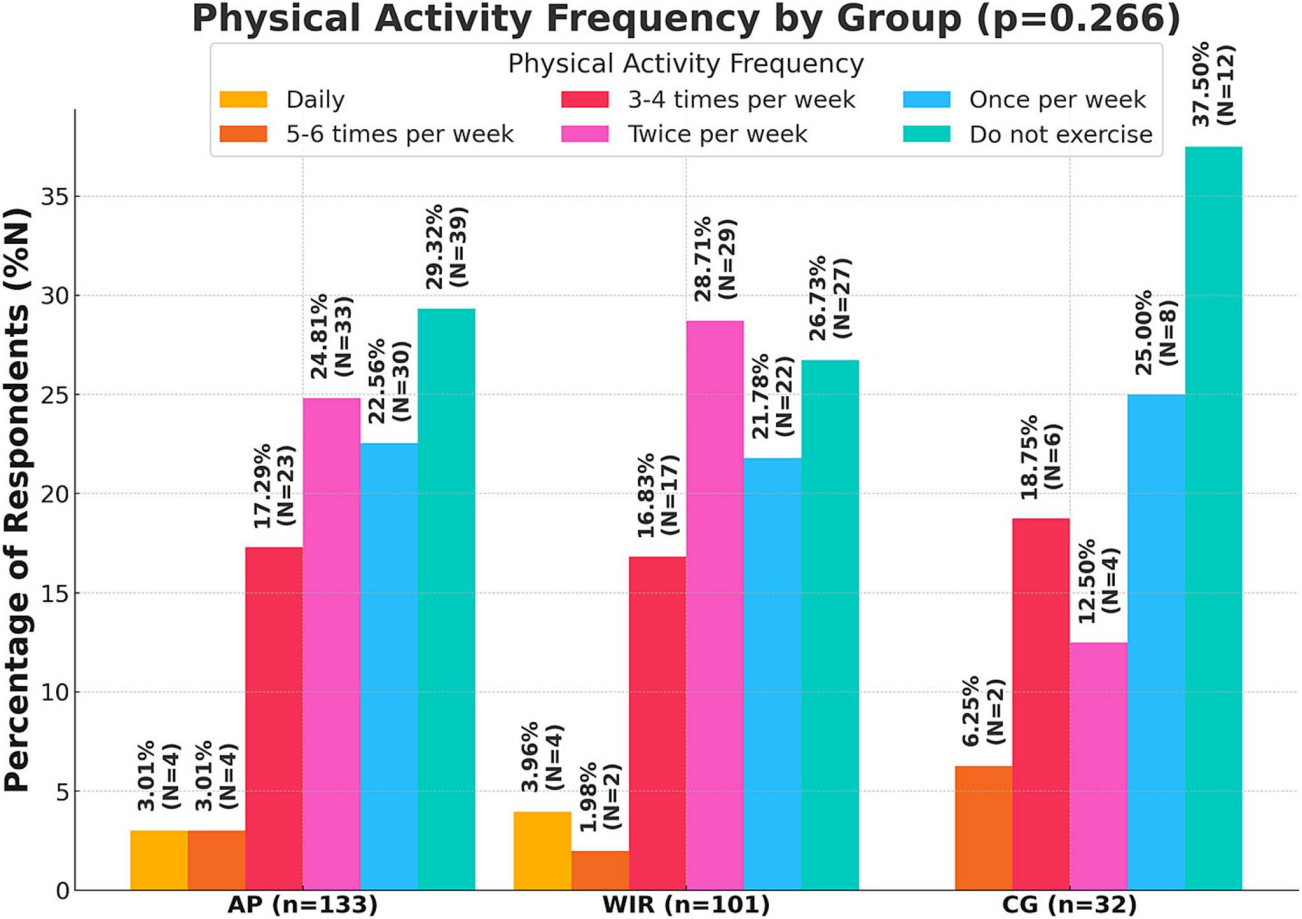
Declared frequency of physical activity per week by respondents (*N* = 133); AP, All Participants; WIR, women with insulin resistance; CG, control group.

Physical activity weekly was practiced twice a week by the majority of women with IR (28.71%; *N* = 29), while the largest number of healthy women did not exercise (37.50%; *N* = 12). There were no statistically significant differences between the groups (*p* = 0.266) Figure 1 shows the details.

### 3.2 BMI of participants

**Figure 2:**
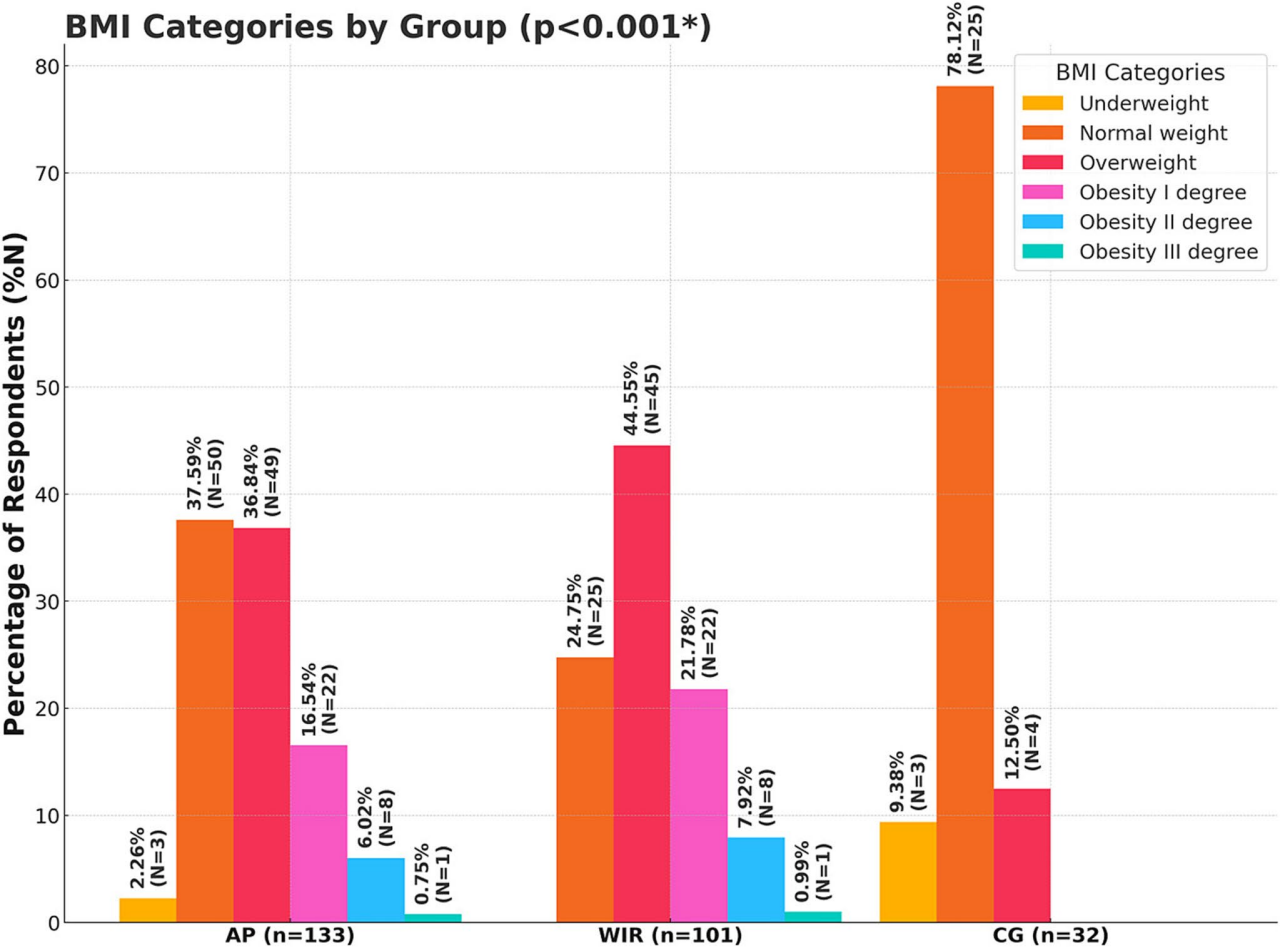
Nutritional status of the subjects expressed through BMI interpretations (*N* = 133); AP, All Participants; WIR, women with insulin resistance; CG, control group; *, *p* < 0.05.

The analysis of BMI revealed significant differences between healthy women and women with IR (*p* < 0.001). Among healthy women, the average BMI was 22.12 ± 3.01, with 78.13% classified as normal weight, 12.50% as overweight, and 9.38% as underweight; none of the healthy women fell into any obesity category. Conversely, women with IR had an average BMI of 28.30 ± 5.16, where 44.55% were classified as normal weight, 24.75% as overweight, 21.78% as obesity I degree, 7.92% as obesity II degree, 0.99% as obesity III degree, and 0.99% as underweight. Finally, women in the AP group had an average BMI of 27.68 ± 4.95, with 37.59% classified as normal weight, 36.84% as overweight, 16.54% as obesity I degree, 6.02% as obesity II degree, 0.75% as obesity III degree, and 2.26% as underweight (Figure 2).

### 3.3 Nutrition knowledge

The table highlights notable differences in dietary knowledge between women with IR and healthy women. Across nearly all categories, the WIR group demonstrated significantly higher knowledge. One of the largest disparities was observed in understanding how to lower the glycaemic load of a salad, with 87.13% of WIR participants answering correctly compared to 37.50% of CG participants (*p* < 0.001). Knowledge of products with a low glycaemic index was also much higher in the WIR group (65.35%) than in the CG group (31.25%, *p* < 0.001). Similarly, the ability to identify the correct type of flour recommended for insulin resistance was significantly greater among WIR participants (69.31%) compared to CG participants (34.38%, *p* < 0.001). Detailed information is presented in Table 2.

**Table 2:** Percentage of correct answers to the questionnaire by group (*N* = 133).

Question	AP *N* = 133 *N* (%)	WIR *N* = 101 *N* (%)	CG *N* = 32 *N* (%)	*p*-value
Definition of insulin resistance	81 (60.91)	70 (69.30)	11 (34.37)	<0.001*
Symptoms of insulin resistance	113 (85.11)	94 (93.07)	19 (59.38)	<0.001*
Determination of glycaemic index	89 (66.92)	76 (75.25)	13 (40.62)	<0.001*
Determination of glycaemic load	41 (30.83)	36 (35.64)	5 (15.63)	0.033*
Low glycaemic index values	76 (57.14)	66 (65.35)	10 (31.25)	<0.001*
Low glycaemic index products	96 (72.93)	83 (82.18)	13 (40.62)	<0.001*
Lowering the glycaemic load of a salad	100 (75.94)	88 (87.13)	12 (37.50)	<0.001*
Correct version of breakfast	97 (72.18)	84 (83.17)	13 (40.62)	<0.001*
Correct version of lunch	123 (92.48)	98 (97.02)	25 (78.10)	<0.001*
Reduced glucose and insulin fluctuations	77 (57.91)	68 (67.33)	9 (28.12)	<0.001*
Type of drinks to limit	121 (90.98)	96 (95.00)	25 (78.12)	0.004*
Type of banana with the lowest glycaemic index	108 (81.20)	85 (84.19)	23 (71.95)	0.121
Type of flour recommended for insulin resistance	81 (60.90)	70 (69.31)	11 (34.38)	<0.001*
Disease resulting from untreated insulin resistance	103 (77.44)	86 (85.15)	17 (53.12)	<0.001*
Familiarity with the concept of ON	51 (38.34)	38 (37.62)	13 (40.63)	0.761

AP, All Participants; WIR, women with insulin resistance; CG, control group; *, *p* < 0.05.

A statistically significant relationship was found between dietary knowledge and the presence of a tissue IR disorder. Knowledge was higher in women with IR than in healthy women (*p* < 0.01). Excellent nutritional knowledge was present in 55.45% (*N* = 56) of women with IR and 25.00% (*N* = 8) of healthy women. Sufficient nutritional knowledge was present among 41.58% (*N* = 42) of women with IR and 21.87% (*N* = 7) of healthy women. Insufficient nutritional knowledge was found in 2.97% (*N* = 3) of women with IR and 53.12% (*N* = 17) of healthy women (Table 3).

**Table 3:** Nutrition knowledge status concerning the groups surveyed (*N* = 133).

Nutritional knowledge	AP *N* = 133 *N* (%)	WIR *N* = 101 *N* (%)	CG *N* = 32 *N* (%)	*p*-value
Insufficient nutritional knowledge	20 (15.04)	3 (2.97)	17 (53.12)	VC = 0.60
Sufficient nutritional knowledge	49 (36.84)	42 (41.58)	7 (21.87)	*F* = 41.40
Excellent nutritional knowledge	64 (48.12)	56 (55.45)	8 (25.00)	*p* < 0.01*

AP, All Participants; WIR, women with insulin resistance; CG, control group; VC, Cramér’s V coefficient; *F*, Fisher’s exact test; *, *p* < 0.05.

### 3.4 Risk of ON

Almost 50% of all women interviewed had a risk of orthorexic behavior (46.62%; *N* = 62). Statistically significant correlations were found between women with or without IR and the risk of ON. In the group of women with insulin resistance, 56.44% (*N* = 57) of respondents were at risk of developing ON, while for healthy women it was 15.63%; (*N* = 5) of respondents (*p* < 0.01). Detailed information is presented in Table 4.

**Table 4:** Risk of ON in study groups (*n* = 133).

Group	Risk of ON (<35 points) *N* (%)	No Risk of ON (>35 points) *N* (%)	*p*-value
AL *N* = 133	62 (46.62)	71 (53.38)	Φ-Yule = 0.34 Chi^2^ = 16.26 *p* < 0.01*
WIR *N* = 101	57 (56.44)	44 (43.56)	
CG *N* = 32	5 (15.63)	27 (84.37)	

AP, All Participants; WIR, women with insulin resistance; CG, control group; Φ-Yule, Φ-Yule coefficient; Chi^2^, Chi-squared test; *, *p* < 0.05.

**Figure 3:**
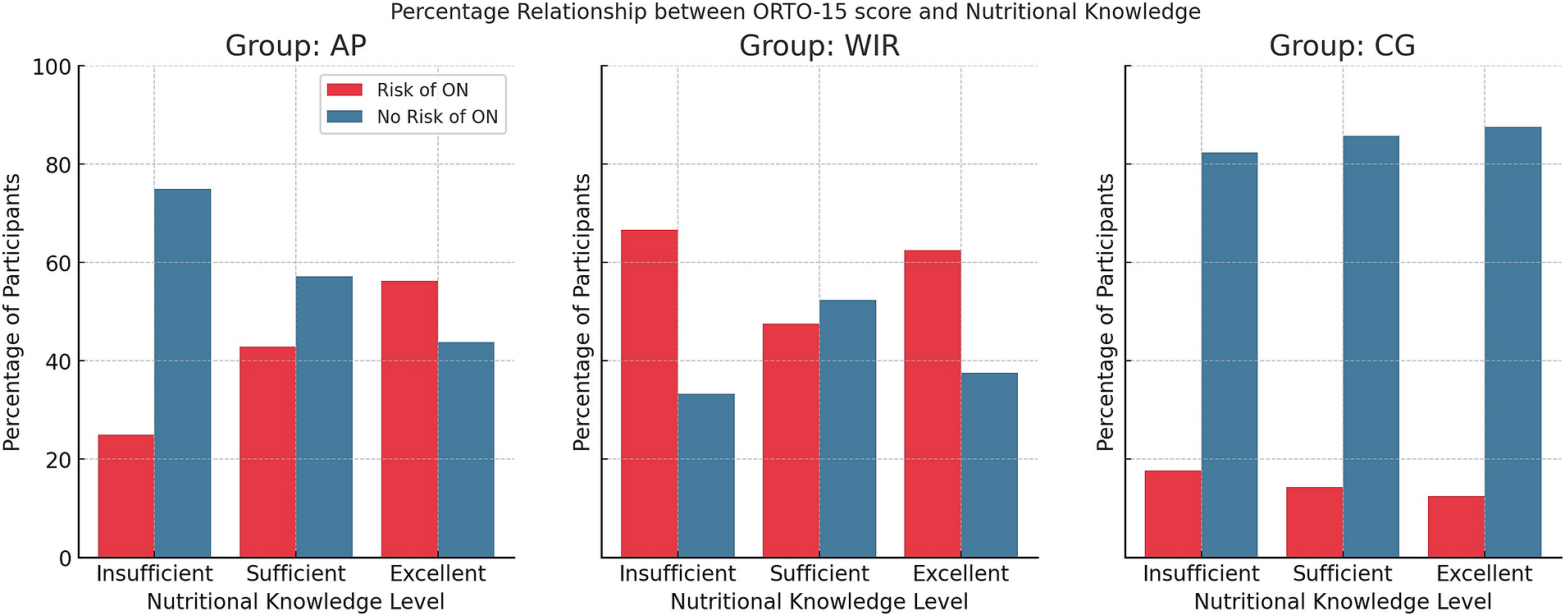
Percentage distribution of ON risk according to level of nutritional knowledge (*N* = 133); AP, All Participants; WIR, women with insulin resistance; CG, control group.

The analysis of nutritional knowledge and ON risk revealed significant differences among groups. In the all participants group, 3.76% (*n* = 5) of individuals at risk of ON demonstrated insufficient nutritional knowledge, 15.79% (*n* = 21) had sufficient knowledge, and 27.07% (*n* = 36) showed excellent knowledge. In contrast, among those without ON risk, 11.28% (*n* = 15) exhibited insufficient knowledge, while 21.05% (*n* = 28) had both sufficient and excellent knowledge. Among women with insulin resistance, 1.98% (*n* = 2) of those at risk of ON showed insufficient nutritional knowledge, 19.80% (*n* = 20) had sufficient knowledge, and 34.65% (*n* = 35) demonstrated excellent knowledge. For women with insulin resistance, without ON risk, 0.99% (*n* = 1) had insufficient knowledge, 21.78% (*n* = 22) sufficient, and 20.79% (*n* = 21) excellent knowledge. In the healthy women group, 9.38% (*n* = 3) of those at risk of ON had insufficient nutritional knowledge, while 3.12% (*n* = 1) had both sufficient and excellent knowledge. Among those not at risk, 43.75% (*n* = 14) demonstrated insufficient knowledge, 18.75% (*n* = 6) sufficient knowledge, and 21.88% (*n* = 7) excellent knowledge. These results highlight a statistically significant relationship between orthorexia risk and nutritional knowledge among all participants but not within specific subgroups. Details are presented in Table 5 and Figure 3.

**Table 5:** Relationship between ORTO-15 score and Nutritional knowledge.

Group	ORTO-15	Insufficient nutritional knowledge	Sufficient nutritional knowledge	Excellent nutritional knowledge	*p*-value
AP *N* = 133	Risk of ON (<35 points)	5 (3.76%)	21 (15.79%)	36 (27.07%)	VC = 0.22
	No Risk of ON (>35 points)	15 (11.28%)	28 (21.05%)	28 (21.05%)	*p* = 0.04*
WIR *N* = 101	Risk of ON (<35 points)	2 (1.98%)	20 (19.80%)	35 (34.65%)	VC = 0.15
	No Risk of ON (>35 points)	1 (0.99%)	22 (21.78%)	21 (20.79%)	*p* = 0.32
CG *N* = 32	Risk of ON (<35 points)	3 (9.38%)	1 (3.12%)	1 (3.12%)	VC = 0.06
	No Risk of ON (>35 points)	14 (43.75%)	6 (18.75%)	7 (21.88%)	*p* = 0.94

AP, All Participants; WIR, women with insulin resistance; CG, control group; VC, Cramér’s V coefficient; *, *p* < 0.05.

### 3.5 Linear regression analysis of predictors of ORTO-15 scores

The linear regression analysis evaluated the relationship between dietary knowledge, BMI, self-reported insulin resistance diagnosis, and ORTO-15 scores. The model showed moderate explanatory power, with an *R* value of 0.597 and an *R*^2^ of 0.357.

Dietary knowledge was a significant predictor of ORTO-15 scores (*p* < 0.001), suggesting that greater dietary knowledge is associated with a higher risk of orthorexia. The presence of IR was also a significant predictor, with individuals diagnosed with IR showing a lower ORTO-15 score (*p* < 0.001), indicating a higher risk of orthorexia. In contrast, BMI did not significantly predict ORTO-15 scores (*p* = 0.810), suggesting no meaningful relationship between BMI and orthorexia risk in this model. Details are presented in Table 6.

**Table 6:** Linear regression analysis of ORTO-15 score and nutrition knowledge scores and BMI with IR diagnosis (*N* = 133).

		Model coefficients – ORTO-15 score; *R* = 0.533; *R*^2^ = 0.285				
Predictor	Estimate	SE	95% confidence interval		*t*	*p*-value
			Lower boundary	Upper boundary		
Intercept	41.7385	2.725	36.347	47.130	15.317	< 0.001*
Knowledge	−0.6361	0.145	−0.923	−0.349	−4.382	< 0.001*
BMI	0.0250	0.104	−0.180	0.230	0.241	0.810
IR Diagnose						
Yes - No	−5.5847	1.469	−8.492	−2.678	−3.801	< 0.001*

SE, Standard Error; *t*, *t*-value; * = *p* < 0.05.

## 4 Discussion

The aim of this study was to assess the level of nutritional knowledge among women with IR and to evaluate the relationship between heightened nutritional awareness and the risk of developing ON. Given the increasing prevalence of IR and the importance of dietary interventions in its management, the study sought to explore whether the pursuit of healthy eating could inadvertently lead to disordered eating patterns, such as ON.

In this study, we employed the ORTO-15 questionnaire to assess the risk of ON. Although the ORTO-15 has been criticized for potentially overestimating ON risk due to its low specificity and concerns regarding validity, the Polish version of the ORTO-15 has been validated specifically for Polish women. The cutoff points established in this validation have been shown to be appropriate for this population, and therefore, our analysis is limited to these adopted cutoff values (20, 21). While alternative cutoff scores, such as those used in the ORTO-11 or ORTO-R versions, might yield different prevalence estimates in other settings, the cutoffs used in our study are optimal for our cohort (21).

The findings of this study suggest that women with IR who exhibit higher nutritional knowledge may also face a greater susceptibility to developing ON. This result is noteworthy as it highlights a complex interplay between the pursuit of optimal dietary patterns, heightened health literacy, and the emergence of potentially pathological eating behaviors.

Previous research has consistently shown that individuals with IR are encouraged to adopt healthier eating habits to improve insulin sensitivity, maintain proper glycemic control, and manage body weight (22). Dietary interventions, including reductions in refined carbohydrates and increases in fiber, lean protein, and nutrient-dense foods, are commonly recommended to these patients (23). Such interventions naturally raise nutritional knowledge, as patients become more informed about dietary composition, meal planning, and nutrient quality. While greater knowledge and adherence to dietary guidelines generally lead to better metabolic outcomes (22, 23), our findings suggest that this knowledge may simultaneously foster rigid attitudes and overconsciousness about food choices—a hallmark of ON.

The analysis showed that women with IR had significantly higher BMI values (28.30 ± 5.16) compared to healthy women (22.12 ± 3.01, *p* < 0.05). These results are consistent with the well-documented relationship between IR and increased body weight, especially obesity. In a study by Stańczyk et al. (24) a similar issue was observed among women with IR and PCOS. Out of the group of 76 respondents, the majority were overweight, with a mean BMI of 27.01 ± 6.11 kg/m^2^, and obesity occurred more frequently than overweight (24). A high BMI is a key risk factor for the progression of insulin resistance, highlighting the importance of early nutritional interventions and lifestyle changes in preventing metabolic complications (25).

The analysis of physical activity revealed concerning trends among healthy women, with 37.50% not engaging in any form of physical exercise. In contrast, the majority of women with IR exercised at least twice a week (28.71%), which may be attributable to medical recommendations aimed at controlling body weight and improving insulin sensitivity in tissues. In the study by Mirska et al. (26) which included 93 adults diagnosed with type II diabetes, 11.8% engaged in physical activity twice a week, 25.8% exercised daily, and 14% did not engage in any form of activity over a week. These findings demonstrate that although Poles with type II diabetes are aware of the importance of physical activity, only about 50% reported exercising regularly (26). The lack of physical activity in the group of healthy women is alarming, given that regular exercise is one of the key elements in the prevention of metabolic disorders.

In terms of nutritional knowledge, women with IR demonstrated a significantly higher level of knowledge compared to the control group. A very high level of nutritional knowledge was noted in 55.45% of women with insulin resistance, whereas only 25.00% of healthy women achieved this level. Conversely, inadequate knowledge was far more common among healthy women (53.12%) than among those with IR (2.97%).

In a study by Pazderska (27), it was found that among 53 individuals living with diabetes, 92.5% knew the principles of a proper diet. However, 62% of these individuals, despite having a very good understanding, did not adhere to these principles, often including contraindicated foods in their diet (27). Our results resonate with previous literature indicating that individuals who are highly informed and vigilant about their eating habits may inadvertently become overly restrictive, eventually increasing their risk of ON (28, 29). These differences highlight the crucial role of nutrition education and dietary consultations following an IR diagnosis. Access to specialized care and dietary recommendations significantly contributes to increasing knowledge among individuals coping with this condition.

The study also demonstrated a significant risk of ON, particularly among women with insulin resistance. More than half of the women with IR (56.44%) were at risk of developing ON, compared to 15.63% of healthy women (*p* < 0.05). The high ON risk in the insulin-resistant group may stem from increased dietary control and a heightened focus on healthy eating following diagnosis. While high nutritional knowledge is generally beneficial, it can also lead to overly restrictive or obsessive eating behaviors.

In a study conducted by Plichta and Jezewska-Zychowicz (30), eating habits were examined in conjunction with the risk of ON and the presence of ED symptoms. The analysis included 1,120 Polish participants, of whom 70.4% were women and 29.6% were men. Approximately half of the respondents were studying health-related fields such as dietetics, food technology, and human nutrition, while the other half were enrolled in non-health-related programs. It was found that 28.3% of the surveyed population tended ON, and half of these individuals also showed ED symptoms. The results indicated a higher ON risk among those who demonstrated positive eating habits—regular meals, consuming four or more meals per day, and reducing sugar and salt intake. Additionally, students following specialized diets also had a higher propensity for ON (30).

Another study by Shoemaker (31), analyzing the occurrence of ON-indicative symptoms, involved 95 individuals with type I and type II diabetes residing in the United States. Participants were surveyed using the ORTO-15 test with a cutoff score of 40. Among those with type I diabetes, 66.7% were at risk of ON, while 62% of those with type II diabetes were at risk. The average ORTO-15 score was 37.4 points. Although the risk of ON was not significantly correlated with eating habits, it appeared more frequently among younger individuals (31). These findings underscore the need to promote a balanced approach to nutrition, focusing on health while avoiding extreme behaviors.

It is important to acknowledge that not all individuals with high nutritional knowledge develop ON. Many people successfully integrate dietary recommendations into a balanced lifestyle without becoming excessively restrictive. Therefore, future research should focus on identifying protective factors—such as psychological resilience, supportive counseling, and balanced nutrition education—that can help maintain a healthy relationship with food while achieving metabolic control (32, 33).

While the primary focus of this study was on women with IR, the findings from the control group of healthy women provide valuable insights into potential protective factors against ON. The control group, despite demonstrating significantly lower levels of nutritional awareness, exhibited a markedly lower risk of developing ON (15.63%) compared to women with IR (56.44%). This discrepancy suggests that lower nutritional vigilance may, paradoxically, act as a buffer against the development of restrictive eating patterns.

One possible explanation for this is that healthy women without IR may experience less pressure to adhere to rigid dietary regimes, allowing for greater dietary flexibility and reducing anxiety around food choices. Unlike women with IR, whose dietary behaviors are often shaped by medical advice emphasizing strict control over food intake, healthy women are less likely to engage in obsessive tracking of nutritional content. This aligns with previous research suggesting that the absence of health-related anxiety and the presence of more relaxed attitudes toward eating contribute to a lower risk of disordered eating behaviors.

Although a high level of nutritional knowledge may facilitate proper dietary management, it is not necessarily problematic by itself. It appears that rigid cognitive patterns regarding food—manifested as an obsessive focus on food quality, inflexible adherence to dietary rules, and an excessive pursuit of the ‘ideal’ diet—play a more critical role in the development of ON. Bratman and Knight (11) demonstrated that an excessive preoccupation with healthy eating can lead to pathological eating behaviors. Similarly, Donini et al. (21) observed that, in individuals with a strong orientation toward healthy eating, it is not merely the accumulation of nutritional information but rather cognitive rigidity and personality traits such as perfectionism and high health anxiety that may predispose them to ON. Moreover, findings by Plichta and Jeżewska-Zychowicz (30) suggest that orthorexic tendencies are significantly associated with perfectionistic traits, further indicating that the inflexible cognitive framework around food might be the driving factor behind ON rather than high nutritional knowledge per se. Additionally, Cena et al. (28) highlight the pivotal role of cognitive inflexibility and anxiety in the manifestation of ON symptoms, underscoring the need for future research to include these psychological variables. In our study, the nutritional knowledge questionnaire primarily assessed factual understanding, without capturing cognitive rigidity or personality factors; thus, the observed association between higher nutritional knowledge and ON risk may be, at least in part, mediated by these underlying factors.

Beyond the realm of dietary education, psychological factors such as health anxiety, perfectionism, and obsessive tendencies may also contribute to the heightened risk of ON among women with insulin resistance. Individuals with metabolic disorders often experience increased concern about their health, which can manifest as rigid control over food choices in an attempt to mitigate perceived risks (18). Perfectionism and obsessive-compulsive traits, in particular, have been shown to correlate with disordered eating patterns, as they can intensify the need to adhere strictly to self-imposed dietary rules (16, 34). Furthermore, the psychological burden of managing a chronic condition like IR may exacerbate stress and anxiety, thereby fueling a cycle in which the individual’s focus on ‘healthy eating’ becomes increasingly inflexible. These findings align with broader literature linking metabolic disorders to mental health conditions, including anxiety and depression, suggesting that the interplay between physiological and psychological factors may be key to understanding how high nutritional knowledge intersects with the development of orthorexic tendencies (35). Future research should therefore integrate measures of psychological distress, perfectionism, and health anxiety to better elucidate the mechanisms by which metabolic challenges and dietary vigilance converge to elevate ON risk.

Given the clinical implications of our findings, it is imperative that healthcare professionals adopt a multifaceted approach when counseling patients with IR to prevent the development of ON. Dietitians should provide sound nutritional education while emphasizing dietary flexibility and intuitive eating, cautioning against overly rigid food rules and obsessive monitoring of dietary intake. Endocrinologists are encouraged to collaborate closely with dietitians by incorporating brief assessments of patients’ mental well-being into routine follow-ups, using standardized checklists that include queries about dietary rigidity and food-related anxiety. Meanwhile, mental health professionals should integrate psychological screening tools—such as the Eating Disorder Examination Questionnaire (EDE-Q), the Hospital Anxiety and Depression Scale (HADS), or the Cognitive Flexibility Scale—into IR dietary counseling to identify early signs of perfectionism, obsessive tendencies, or health anxiety, and to facilitate timely referrals for psychological interventions (36–38). Such interdisciplinary collaboration is essential to balance the benefits of dietary education with the need to prevent excessive dietary vigilance, thereby supporting both the metabolic and mental health of patients.

### 4.1 Strengths and limitations

Strengths of this study include a well-defined study population, which allowed a direct comparison between women with IR and healthy women, as well as the use of the standardized ORTO-15 questionnaire. The research approach including BMI, dietary knowledge, physical activity and ON risk provided a multifaceted perspective on the interplay between metabolic health and eating behavior. However, several limitations should be noted. Reliance on self-reported data may introduce bias or inaccuracies. It should be emphasized that the cross-sectional nature of our study does not allow for the establishment of a causal relationship between nutritional knowledge and the risk of developing ON. Although our findings indicate a significant association between these variables, future longitudinal studies or interventional trials are necessary to determine whether an increase in nutritional knowledge contributes to the development of ON or if individuals who are predisposed to ON are more inclined to seek out additional dietary knowledge. Moreover, the study was conducted in a single geographical and cultural context with a relatively uniform socioeconomic level, which may affect participant behaviors and limit the generalizability of the findings to other populations. Additionally, we did not broadly account for psychosocial variables (such as perfectionism and health anxiety) that may influence both eating behavior and risk of ON. Our study also focused exclusively on women, providing no insight into how insulin resistance and nutritional awareness might influence orthorexic tendencies in men. This represents a potential area for future research, as examining the experiences of men with IR could offer a more comprehensive understanding of gender differences in the development of ON and other disordered eating patterns. Furthermore, psychiatric diagnoses were excluded based on medical documentation and physician-conducted interviews, without direct consultation with a psychiatrist. No standardized diagnostic tools for mental disorders were used, which may limit the accuracy of psychiatric exclusions. This lack of differential diagnosis for ON is an additional limitation of the study.

## 5 Conclusion

The results of this study suggest that women with IR, despite demonstrating higher levels of dietary knowledge and efforts to adhere to dietary recommendations, are at increased risk of developing ON. While their dietary awareness may support better metabolic control, it also appears to contribute to rigid and overly restrictive eating behaviors. In contrast, the healthy women in the control group exhibited lower dietary knowledge, which, although potentially linked to less optimal eating habits, did not translate into an elevated ON risk. Due to the cross-sectional design of this study, causality between increased nutritional knowledge and the risk of ON cannot be confirmed. Therefore, we recommend that future research include longitudinal studies and interventional trials to better understand the directionality of this relationship.

These findings highlight the need for healthcare providers to adopt a more balanced and psychologically sensitive approach to dietary counseling for women with IR. Training programs aimed at equipping dietitians and general practitioners with the skills to identify early signs of ON could be instrumental in preventing the development of disordered eating patterns. Integrating psychological assessments into routine dietary consultations may help detect perfectionism, anxiety, or rigid attitudes toward food, allowing for timely intervention. Collaboration between dietitians, psychologists, and endocrinologists is essential to provide comprehensive care that addresses both the physical and mental health needs of patients. Emphasizing dietary flexibility, intuitive eating, and fostering a positive relationship with food during counseling sessions can reduce the likelihood of restrictive behaviors. By promoting moderation and addressing the psychological dimensions of dietary adherence, healthcare professionals can help women with IR manage their condition without compromising their mental.

## Data Availability

The original contributions presented in the study are included in the article/supplementary material, further inquiries can be directed to the corresponding author.
